# Crop phenotype prediction using biclustering to explain genotype-by-environment interactions

**DOI:** 10.3389/fpls.2022.975976

**Published:** 2022-09-20

**Authors:** Hieu Pham, John Reisner, Ashley Swift, Sigurdur Olafsson, Stephen Vardeman

**Affiliations:** ^1^Department of Information Systems, Supply Chain, and Analytics, College of Business, The University of Alabama in Huntsville, Huntsville, AL, United States; ^2^Department of Statistics, Iowa State University, Ames, IA, United States; ^3^Department of Industrial and Manufacturing Systems Engineering, Iowa State University, Ames, IA, United States

**Keywords:** linear model, no-interaction model, missing data, unsupervised learning, machine learning

## Abstract

Phenotypic variation in plants is attributed to genotype (G), environment (E), and genotype-by-environment interaction (GEI). Although the main effects of G and E are typically larger and easier to model, the GEI interaction effects are important and a critical factor when considering such issues as to why some genotypes perform consistently well across a range of environments. In plant breeding, a major challenge is limited information, including a single genotype is tested in only a small subset of all possible test environments. The two-way table of phenotype responses will therefore commonly contain missing data. In this paper, we propose a new model of GEI effects that only requires an input of a two-way table of phenotype observations, with genotypes as rows and environments as columns that do not assume the completeness of data. Our analysis can deal with this scenario as it utilizes a novel biclustering algorithm that can handle missing values, resulting in an output of homogeneous cells with no interactions between G and E. In other words, we identify subsets of genotypes and environments where phenotype can be modeled simply. Based on this, we fit no-interaction models to predict phenotypes of a given crop and draw insights into how a particular cultivar will perform in the unused test environments. Our new methodology is validated on data from different plant species and phenotypes and shows superior performance compared to well-studied statistical approaches.

## 1. Introduction

Plant phenotypes, such as flowering time or yield, depend on a plant's genotype and the environment where it is grown. However, a plant's phenotype is typically not well-explained by simple main effects of genotype (G) and environment (E), as there is often important genotype-by-environment interaction (GEI) effects that explain a considerable portion of the observed phenotype variation. Understanding the GEI effects, and accounting for such effects in any predictive model, is therefore of great interest to plant breeders and agronomists who aim to develop new genotypes that have a favorable phenotypic trait and/or performance across diverse environments (Ahakpaz et al., 2021; Ligarreto-Moreno and Pimentel-Ladino, 2022).

Numerous predictive models of phenotype have been proposed in the literature (Asseng et al., 1998; Chuine et al., 1999; Lobell and Burke, 2010; Zhou et al., 2012). Most recent models improve predictions by incorporating various environmental and genetic information (Montesinos-López et al., 2019; Arya et al., 2022; Nguyen et al., 2022), but in some applications only phenotype data may be available, and a model must be built exclusively on this data. For our analysis, we assume that this is the case, including that the only available data are phenotypes observed for a set of genotypes in a set of environments. A model must therefore be built on a simple two-way table of phenotype where rows represent genotype and columns represent environments. Furthermore, phenotypes may have only been observed in relatively few environments for each genotype, leading to a two-way table where many of the values are missing. This is common in commercial plant breeding where it is either impractical or impossible to observe the genotype except in a few environments. Thus, the majority of the potential phenotype observations are missing. Furthermore, since the tested environments depend on such factors as decisions of plant breeders, observations are missing not-at-random in an unknown fashion. This scenario is the motivation for our new methodology, which aims to model phenotype based on a two-way table of observed phenotypes with the potential for most of the values in the table to be missing according to some unknown non-random mechanism.

Simple approaches for modeling phenotype are taken to either ignore the GEI effects or incorporate all interaction terms directly into a linear model for the phenotype. The first approach completely missed practically important interactions. The latter approach produced unique estimates (and clear interactions) for all main effects and interactions only when no values are missing. When values are missing, a model containing all interactions is equivalent to a “cell means model” and provides no way to predict missing values from data in hand. Both extreme modeling solutions are typically unsatisfactory, which has motivated the development of several alternative models that aim to both have fewer parameters and provide modeling for GEI effects. Malosetti et al. (2013) surveyed the state-of-the-art for such models where only a two-way tables of means were given including that each model attempted to replace the GEI term with some combination of genotype and environmental parameters.

In this paper, we propose a novel approach to modeling and explaining GEI effects by identifying useful subsets of the genotypes and environments *via* an approach called biclustering. Biclustering simultaneously clusters rows (which here represent different plant genomes) and columns (which here represent different planting environments). By choosing an appropriate response function, the biclustering can produce homogeneous blocks of genotypes and environments where each genotype interacts in the same manner with the environments. This implies (locally) that, within each such block there are in fact no GEI interaction effects and a linear model employing only main effects (G and E) provides an appropriate fit for the phenotype. While our analysis only uses phenotypes as responses, further information about the genotypes and/or the environments in a block can be used after the fact to explain and provide insights into commonalities of genetics and of environments.

Although similar biclustering approaches have been utilized to explain interactions in a two-way table in various domains, these methods assume complete data, that is, no missing values (Schepers et al., 2017). Moreover, in regards to understanding GEI interactions, Corsten and Denis (1990) performed a study by applying agglomerative hierarchical clustering to a two-way table to analyze the interaction between rows (genotypes) and columns (environments). However, one limitation of their work is their assumption on having complete data. For an academic trial, the assumption of complete data may hold, but for a commercial large scale breeding organization, missing data is the norm. Missing values pose a major obstacle for standard biclustering methods. Partially motivated by this problem, Li et al. (2020) proposed a new biclustering method that performs well even for highly incomplete datasets (where many values are missing). For our purposes, since we want a methodology that works for phenotype data with missing values, we use the biclustering method of Li et al. (2020). Specifically, our work contributes to the literature as follow.

- We apply a novel biclustering approach to models G and E. This is the first paper to consider modeling crop phenotypes with this methodology.
- Our approach is effective in the case of missing data whereas other studies either impute or remove missing observations.

After presenting the details of the new methodology, we apply our approach to three phenotype datasets. We analyze data on three different crops (sorghum, maize, and rice) and two phenotypes (flowering time and yield). Each of these cases illustrates different aspects of our new methodology, and we compare its performance to that of existing methods. Moreover, we utilize these datasets as an opportunity to display more detailed workings of our methodology with respect to the interpretation of visualizations and numerical results.

## 2. Methodology

In this section, we describe the details of the new methodology. We first describe the main idea of using a set of no interaction models to model phenotype, and then present a specific method for obtaining those sets *via* biclustering.

### 2.1. Modeling interactions

As noted in the introduction, we model plant response or phenotype (μ_*ij*_) for a set of genotypes *i* ∈ *I* in environments *j* ∈ *J*. Without using further explanatory variables to describe either the genotype or the environment, we could model phenotype with a simple no interaction model


$$\mu_{ij}=\mu +G_{i}+E_{j}+\epsilon_{ij}$$

where μ, *G*_*i*_ for *i* ∈ *I*, and *E*_*j*_ for *j* ∈ *J* are unknown constants, and ϵ_*ij*_ is an independent normal error. Here, genetics and environment are not interacting with respect to determining μ_*ij*_. This is typically unrealistic for *I* and *J* of any size and/or widely varying genotypes or environments. Full generality would then require modeling of the form


$$\mu_{ij}=\mu +G_{i}+E_{j}+GEI_{ij}+\epsilon_{ij},$$

for additionally unknown constants *GEI*_*ij*_ for *i* ∈ *I* and *j* ∈ *J*.

Even though not all GEI effects may be important, one can imagine that in some cases there are subsets of genotypes that interact in the same manner with a subset of environments. We let *I*_0_ ⊂ *I* and *J*_0_ ⊂ *J* denote such subsets. In such a case,


$$GEI_{i_{1}j}=GEI_{i_{2}j},\forall i_{1},i_{2}\in I_{0},j\in J_{0}$$

and we will use the notation $GEI_{j}^{0}$ for the value of *GEI*_*ij*_ common across *i* ∈ *I*_0_. Then, for ${Ẽ}_{j}^{0}=E_{j}+GEI_{j}^{0}$ (a “block” environmental main effect)


$$\mu_{ij}=\mu +G_{i}+{Ẽ}_{j}^{0}+\epsilon_{ij},$$

for *i* ∈ *I*_0_ and *j* ∈ *J*_0_. In other words, within the scope of *I*_0_ and *J*_0_, a linear no interaction model is appropriate.

Expanding on this idea, suppose we can partition *I* and *J* into non-overlapping subsets, *I* = *I*_1_ ∪ *I*_2_ ∪ … ∪ *I*_*n*_ and *J* = *J*_1_ ∪ *J*_2_ ∪ … ∪ *J*_*m*_ where *GEI*_*i*_1_*j*_ = *GEI*_*i*_2_*j*_, ∀*i*_1_, *i*_2_ ∈ *I*_*p*_, *j* ∈ *J*_*l*_, *p* = 1, .., *n*; *l* = 1, …, *m* and that within every block {(*i*_*j*_)|*i* ∈ *I*_*p*_, *j* ∈ *J*_*l*_} the *GEI*_*ij*_ depends only upon *j*. Then for $GEI_{j}^{p}$, the value of *GEI*_*ij*_ common across values of *i* ∈ *I*_*p*_ and ${Ẽ}_{j}^{p}=E_{j}+GEI_{J}^{p}$, one has


$$\mu_{ij}=\mu +G_{i}+{Ẽ}_{j}^{p}+\epsilon_{ij}$$

for all *i* ∈ *I*_*p*_ and *j* ∈ *J*_*l*_. That is, *inside of each one of n* × *m* *blocks* of genotypes/rows by environments/columns, there is an additive (“no-interaction”) structure. If such structure exists and can be identified, then there is the possibility of fitting *n* × *m* no-interaction models within blocks and having sensible ways to predict missing values without making the overly stringent global additivity in a linear model for phenotype. We proceed to introduce the methodology for searching a large (and potentially sparse) two-way table of mean responses for such “no-interactions within blocks” structure based on biclustering.

### 2.2. Biclustering

**Figure 1:**
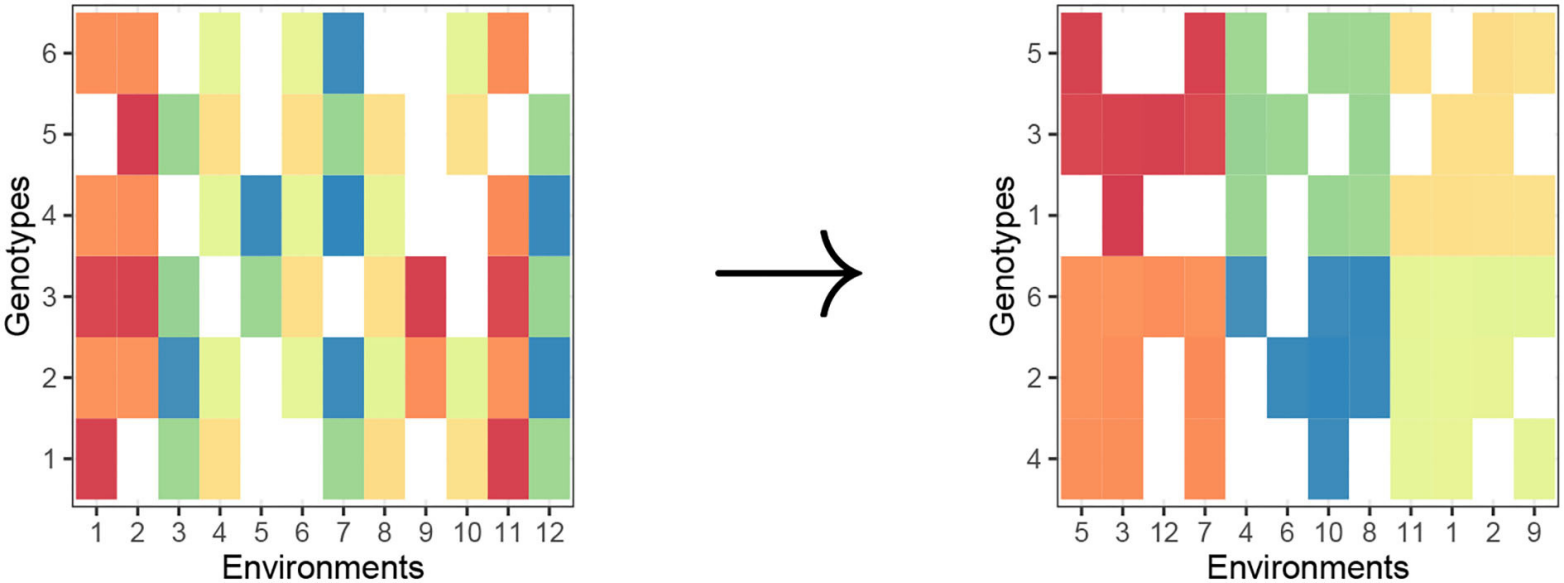
Biclustering creates row clusters (genotypes) and column clusters (environments) with homogeneous phenotype in a checkerboard pattern. Raw matrix **(left)** and “checkerboard-like” biclustering **(right)**.

Biclustering is a statistical learning methodology that clusters both rows and columns in a two-way data tables simultaneously (This is in contrast to traditional one-way clustering methods, such as complete-link hierarchical clustering and *k*−means clustering which partitions only rows.). Since we need partitions of both genotypes and environments, the type of partitioning that we need is biclustering. In Figure 1, we provide a visual of the objective of a general biclustering algorithm. For our purposes, one can consider biclustering where the rows, *i*, represent genotypes and columns, *j*, represent environments. The color in each cell (in a heat map manner) can be interpreted as some phenotype μ_*ij*_ or a transformation of phenotype, for a genotype-environment pair, and the standard objective of biclustering is to make *homogeneous* biclusters (in terms of values in the two-way dataset).

Basic biclustering aims to produce blocks of cells with homogeneous responses. This is not obviously is directly aligned with our ultimate modeling objective. But appropriate pre-processing (before biclustering) of phenotype responses can produce what is ultimately needed. We, therefore, consider biclustering four different possible responses, the phenotype directly (as reference) and three transformations that all normalize the phenotype response as shown in responses (1)–(4).

1. μ_*ij*_ − μ Difference from overall average (direct response)
2. $\mu_{ij}-{\bar{\mu }}_{i}-{\bar{\mu }}_{j}+\mu$ Difference from genotype and environment averages
3. $\mu_{ij}-{\bar{\mu }}_{i}$ Difference from genotype *i* average
4. $\mu_{ij}-{\bar{\mu }}_{j}$ Difference from environment *j* average

The results of biclustering response (1) are most easily interpreted as a direct representation of phenotype. While possible that this results in the desired biclusters, there is no guarantee that this will happen. However, applying biclustering to responses (2)–(4) can in all cases result in the identification of blocks of genotypes and environments with approximately the same GEI effects. To see this, we examine those response functions in more detail. The second response is in fact an expression for the GEI effects directly, as can be shown from a few steps of algebra:


$$\begin{array}{l} y_{ij}=\mu_{ij}-{\bar{\mu }}_{i}-{\bar{\mu }}_{j}+\mu \\ \;=\mu +G_{i}+E_{j}+GEI_{ij}+\epsilon_{ij}-{\bar{\mu }}_{i}-{\bar{\mu }}_{j}+\mu \\ \;=\mu +\left({\bar{\mu }}_{i}-\mu \right)+\left({\bar{\mu }}_{j}-\mu \right)+GEI_{ij}+\epsilon_{ij}-{\bar{\mu }}_{i}-{\bar{\mu }}_{j}+\mu \\ \;=GEI_{ij}+\epsilon_{ij} \end{array}$$

It is therefore immediately clear that clustering the second response should result in cells where the varieties have the same GEI effects and additivity within each cell.

The third response, that is, the deviation from genotype average, is not a direct measure of GEI effects but can be simplified as follows:


$$\begin{array}{l} y_{ij}=\mu_{ij}-{\bar{\mu }}_{i}\\ \;=\mu +G_{i}+E_{j}+GEI_{ij}+\epsilon_{ij}-{\bar{\mu }}_{i}\\ \;=\mu +\left({\bar{\mu }}_{i}-\mu \right)+\left({\bar{\mu }}_{j}-\mu \right)+GEI_{ij}+\epsilon_{ij}-{\bar{\mu }}_{i}\\ \;=\left({\bar{\mu }}_{j}-\mu \right)+GEI_{ij}+\epsilon_{ij} \end{array}$$

This is a sum of the deviation from the environment average and the GEI effects. Clustering and obtaining a perfectly homogeneous cell would thus result in placing genotypes together if this sum is constant. Since this must then be enforced across all column clusters (clusters of environments), it follows that the GEI effects must in fact be constant within each cell. By symmetry of the two-by-two matrix, it is clear that for the fourth response


$$\begin{array}{l} y_{ij}=\left({\bar{\mu }}_{j}-\mu \right)+GEI_{ij}+\epsilon_{ij} \end{array}$$

and the same conclusion holds. While any of the three responses will in principle work, it is not clear which will be most useful in practice. This will be examined empirically later through a series of case studies.

The quality (for our purposes) of a biclustering can be measured in three ways. First, we can consider how well the algorithm performs with its assigned task, namely how homogeneous the response biclustered is within each cell. This will be numerically different for each transformation of the phenotype. Second, we can measure how well it succeeds in finding cells that are homogeneous with respect to estimated (“whole dataset”) GEI effects, which is the desired input for the phenotype model. We note that this is equivalent to the second response being homogeneous. All four transformations (response functions) can be reasonably compared with this approach. Third, we can evaluate the quality of the fit of a no-interaction model within each cell, that is, the output of our modeling approach. The ultimate goal is to obtain a set of cells that result in a no-interaction model being a good fit within each cell, thus this is the most sensible measure of the biclustering quality in our present context.

Due to the complexity of searching through all possible row and column partitionings, biclustering is known to be NP-hard. Because of this, biclustering algorithms generally take a heuristic approach that converges to a local optimal solution. There are numerous biclustering algorithms motivated by gene expression data. For example, Kluger et al. (2003) proposed a method called spectral biclustering which attempts to rearrange a raw data matrix into a “checkerboard-like” structure, such as in Figure 1. Another common biclustering algorithm is by Cheng and Church (2000) which, unlike spectral biclustering, utilizes a node-deletion algorithm to find submatrices already hidden in a two-way data table. However, a limitation to most biclustering algorithms is an inability to effectively handle missing values, especially when there is substantial percentages of missing values that are not missing at random. Instead, most applications of biclustering which involve missing data are handled by an imputation approach (Veroneze et al., 2011; Cheng et al., 2012; Chattopadhyay et al., 2016).

This is critical for our purposes because commercial phenotype data often has a large percentage of the data missing not-at-random, and imputation methods will not be effective in handling such high percentages of missing data. In fact, as noted in the introduction, the initial motivation for our methodology comes from commercial plant breeding, where numerous plant varieties (in the case of soybeans) are selected for advancement, that is, planted at least one more year based on experimental field data. However, each variety is only tested in a small number of environments. This is both due to cost considerations and the suitability of the variety to the environment (e.g., a soybean variety will not be planted in environments that are significant mismatches to it's relative maturity). Thus, the majority of the data is missing not-at-random but based on the year the variety starts trials, relative maturity of the variety, and breeder decisions. Motivated by this and other similar problems, we propose the utilization of a recently discovered biclustering algorithm. This approach to modeling phenotypes makes no assumption about the structure of the data and is still effective with missing data (Li et al., 2020). With this methodology, we are now able to effectively bicluster a two-way table of means where data is missing, and we no longer need to impute values that can bias our data and results. It is therefore Li et al. (2020)'s biclustering algorithm that will be applied in all of the case studies. In this paper, the biclustering algorithm and associated visualizations are created in R using the biclustermd package (Reisner et al., 2019, a companion to Li et al., 2020).

## 3. Results

We apply the proposed methodology to several case studies involving data representing a variety of crops and phenotypes, including data from both university studies and commercial plant breeders (see Table 1).

**Table 1:** Case study characteristics.

**Crop**	**Phenotype**	**Genotypes**	**Environments**	**Missing values**
Sorghum	Flowering time	237	7	3.0%
Maize	Yield	211	8	0%
Rice	Flowering time	176	9	2.8%

For each of the cases, we apply our phenotype model, interpret the results, and compare results to those from other methods that have been proposed in the literature (see Table 2). We limit ourselves to other models that required the same input, that is, a simple two-way table of means for the observed phenotype. Models that take advantage of other explanatory variables are not considered. Models that incorporate such additional information include the factorial regression models (Denis, 1988) and van Eeuwijk et al. (1996) that explicitly incorporate environmental variables and a large number of more recent models that incorporate genotypic information (Pantazi et al., 2016; Chlingaryan et al., 2018; Vitor et al., 2019). In applications where such additional information is available, one of those models is likely to explain more of the GEI effects than a model that uses a simple table of means (Malosetti et al., 2013). Although the maize data do not contain missing values, we included them in our study for completeness. Indeed, we show that even in cases without missing data, our biclustering approach still obtains superior performance.

**Table 2:** Benchmark models for genotype-by-environment interaction (GEI) using a two-way table of phenotype means data.

	**Model type**	**Model**
1.	Additive Model (no-interaction)	μ_*ij*_ = μ + *G*_*i*_ + *E*_*j*_ + ϵ_*ij*_
3.	Regression on the Mean	μ_*ij*_ = μ + *G*_*i*_ + *E*_*j*_ + *b*_*i*_*E*_*j*_ + ϵ_*ij*_
	(see Finlay and Wilkinson, 1963)	
4.	Additive Main Effects and Multiplicative Interactions	$\mu_{ij}=\mu +G_{i}+E_{j}+\overset{K}{\underset{k=1}{\sum }}b_{ik}z_{jk}+\epsilon_{ij}$
	(AMMI) (see Gollob, 1968)	

Moreover, we do not emphasize the percentage of missing values as different field trials may result in varying amounts of missingness. Li et al. (2020) demonstrate that their biclustering algorithm is effective in cases with 75%+ missing values, and we direct the interested reader to their study. Data imputation constructs a complete dataset that enables analysis through traditional linear models, however, it has its limitations. The state at which the data is missing may lead to biased imputed results, that is, data may be missing at random or because of some unknown mechanism. By imputing these results, we are essentially making a claim that we can accurately estimate the phenotype given the variety and environment. For this study, we want to avoid any such claims. Therefore, we do not perform any imputation for comparison. This is the reason we include the maize dataset in our work.

Before we begin our analysis, we note that models (1) and (2), in Table 2, can be seen as special extreme cases of our approach. At one extreme, if we set the number of column and row clusters both equal to one then our model is the same as (1). At the other extreme, if we set the number of row clusters equal to the number of genotypes and the number of column clusters equal to the number of environments, then our model is the same as (2).

### 3.1. Sorghum flowering time data

The first of our cases involve trials studying the flowering time (in growing degree days) of sorghum (*sorghum bicolor*) (Li et al., 2018). The trials were conducted in seven locations (three in Puerto Rico, two in Iowa, and two in Kansas) using 150 recombination inbred lines (RILs) from two inbred parent varieties. In their analysis, the authors use one-dimensional hierarchical clustering to separately cluster varieties and environments and find that by dividing the varieties into two row clusters and environments into three column clusters, the response becomes much more predictable.

#### 3.1.1. Selecting response to biclustering

**Figure 2:**
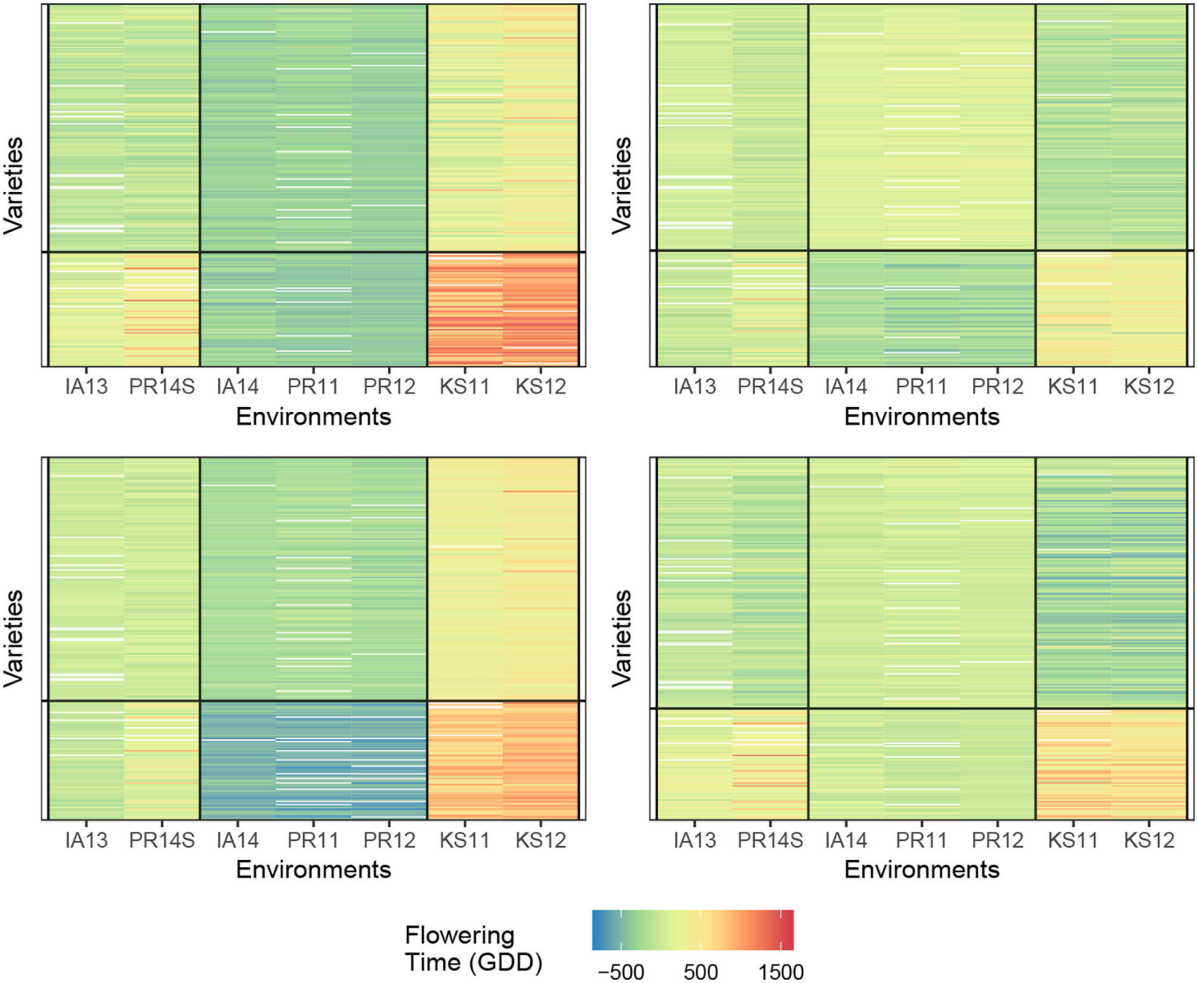
Comparison of biclusterings plots for sorghum. The homogeneity of the cells indicates that the biclustering algorithm was able to successfully group the response variables in distinct clusterings. Top left—response (1); Top right—response (2); bottom left—response (3), and bottom right—response (4).

Determining the number of row and column clusters can be done *via* experimentation as will be described later, but the original study concludes that the environment should be clustered into three clusters, and the genotypes should be clustered into two clusters with the three environmental clusters being {KS11, KS12}, {IA14, PR11, PR12}, and {PR14S, IA13}. Following that conclusion, we start with two-row clusters and three-column clusters. This allows us to move directly into choosing a response function, and we use each of the four response functions described in Equations (1)–(4), with the final biclusters for each of the four response functions is shown in Figure 2. For this dataset, it appears that biclustering every response results in very similar results with respect to creating cells where no-interaction models provide good fits. We believe this to be partially coincidental for the following reason. The environments that show the greatest degree of near additivity, that is, lack of GEI interactions also happen to have similar flowering times when compared to the other environments. This can be seen by the homogeneity of the distinct coloring of cells. For example, locations {IA14, PR11, and PR12} show the absence of GEI interactions and also have fewer days until flowering than the other four locations. While such correlations between the mean response and plasticity may well exist, the fact that we obtain such good biclusters simply by clustering the mean response itself cannot be expected to generalize to more complex datasets with more varieties and environments. As a point of validation, using two genotype clusters and three environment clusters for our biclustering algorithm, we arrive at the same conclusion as Li et al. (2018) with the environmental and genotype clusters coinciding with our biclustering results.

We summarize the performance of each of the biclusters obtained with the measurement being the final within the block sum of squared errors summed across all cells, which we henceforth denote as SSEbc. It should be noted that the biclustering algorithm has a random initialization (the biclustering algorithm randomly assigns genotypes and environments to clusters in the first iteration). Furthermore, the algorithm can converge to a local optimum because of this, successive runs of the algorithm may obtain different results. Using the results from 30 trials, we obtained the smallest SSEbc to be approximately 27 × 10^6^, 36 × 10^6^, 46 × 10^6^ for responses (2), (3), and (4), respectively. Since in practice one would utilize the biclustering algorithm until the best results are obtained, it is reasonable to only report the smallest value acquired.

If we consider the SSEbc as our metric for judging cell homogeneity, we observe that clustering response (2) (GEI directly) obtains the most homogeneous cells, which is the intent of this biclustering algorithm. Whereas, clustering the response directly, response (1), results in the least desirable grouping of varieties and environments. In fact, Li et al. (2018) note that the prediction accuracy depends on first transforming the data from the actual raw number of days until flowering to a modified response that accounts for the amount of solar radiation received in the different locations. Without this transformation and the knowledge that is behind the transformation, the direct hierarchical clustering fails which can be understood by the biclustering response (1). Moreover, we do not include response (1) in these comparisons of “SSE”. The “constant-within-a-cell” SSE is of necessarily bigger than the other responses, and clustering for homogeneous values in a cell has a different objective than the other responses.

#### 3.1.2. Determining number of clusters

In the preceding experiments, we followed the conclusions of Li et al. (2018) and used two-row (genotype) clusters and three-column (environment) clusters. We now examine the ways to determine good numbers of column/row clusters based on the biclustering itself. We do this by considering the two evaluation measures.

a. The response within each cell, that is, SSE_bc_.
b. The fit of a no-interaction model within each cell of the final bicluster, that is, SSE_ni_.

As previously mentioned, the goal of applying biclustering to data such as this is to identify subsets of genotypes and subsets of environments where no-interaction models are appropriate. Once identified, we can fit a no-interaction model $\mu_{ij}=\mu +G_{i}+{Ẽ}_{j}^{p}+\epsilon_{ij}$ for the phenotype within each cell, and then aggregate the sums of squares of each individual model as the measure of performance. From Li et al. (2018), we know that there are two genotype and three environment clusters, respectively, resulting in six cells. Constructing a linear model on the bicluster result, we obtain six linear models with six associated SSE. If we take the sum of the six SSEs, we obtain the final error of our biclustering no-interaction model which we denote as SSE_ni_.

**Figure 3:**
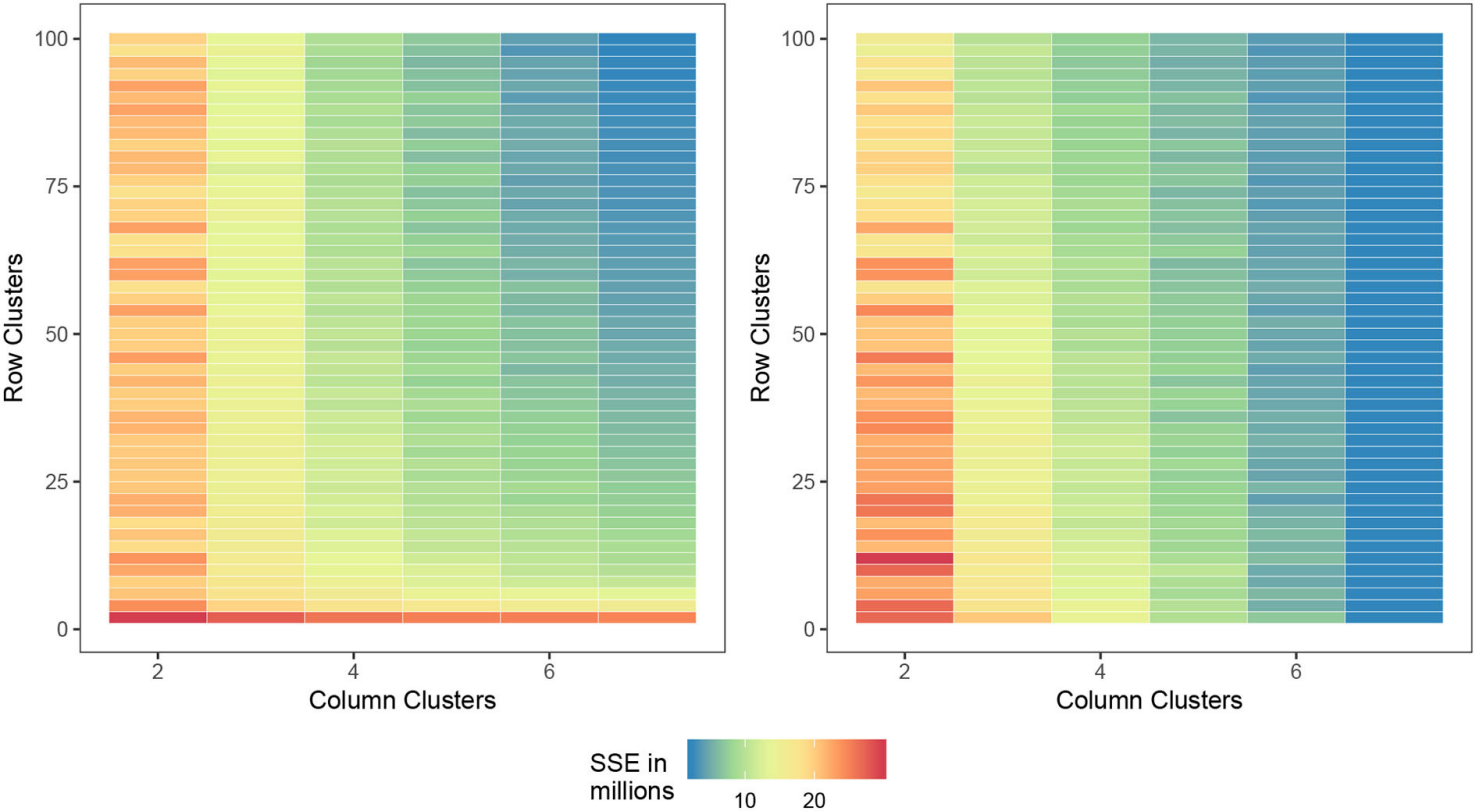
Comparison of the number of row and column clusters for SSEbc **(left)** and SSEni **(right)** as the number of column and row clusters approach the dataset dimensions, the SSE decreases toward zero.

Figure 3 displays the two measures where the cell value is the average SSEbc and SSEni of the (G, E)-pair (i.e., the pair of row and column clusters) over 30 trials. We first notice that as the number of clusters approaches the number of varieties and environments, each observation is placed in its own cell and all of the measures converge to zero, but this is expected since capturing all the GEI interactions will result in zero SSEbc and SSEni. In practice, one would want to avoid cluster pairs on the top right of each figure. If we choose a (G, E)-pair which is slowly transitioning from bottom left (yellow) to top right (green), we can safely choose a combination of row and column clusters while still preserving the information gained from biclustering. Verifying the results of Li et al. (2018), it appears as though two genotype clusters and three environmental clusters are indeed reasonable.

#### 3.1.3. Comparison with other models

We finally compare our modeling approach with the other approaches that only use a two-way table of means for phenotype as input (see Table 2). Namely, we consider linear models 1–4 in Table 2, as our benchmark for the results obtained from biclustering.

The error degrees of freedom and SSE is what is being compared between the linear and biclustering models. Degrees of freedom being equal, a smaller SSE indicates that more error is accounted for within the model. If sums of squares are equal, a higher error degree of freedom would indicate that the model obtains the same quality of fit with less complexity.

Table 3 displays the degrees of freedom and sum of squares for each main effect and other terms. For the no-interaction model in Table 3, we see that there is a large error term due to the variation in the G and E term not being able to capture enough information. However, in the all-interaction model in Table 3, we have no error since all the empirical information is represented by the GEI term. Although this leads to zero SSE in the all interactions model, a linear model with this structure is not useful in our context since this does not provide a straightforward way of separating the GEI from the error. Allowing for interactions at every combination of G and E is not a useful approach to understanding the GEI terms.

**Table 3:** Summary of degrees of freedom and sum of squares for the sorghum dataset.

**Term**	**Degrees of freedom**	**Sum of squares**
**No-interaction model**		
G	236	53,020,738
E	6	199,593,392
Error	1367	67,635,610
**All-interaction model**		
G	236	53,020,738
E	6	199,593,392
GEI	1367	67,635,610
Error	0	0
**Regression on mean model**		
G	236	53,020,738
E	6	199,593,392
GEI Ind	236	52,422,217
Error	1131	15,213,393
**AMMI model**		
G	236	53,020,738
E	6	199,593,392
PC1	241	54,483,854
PC2	239	5,581,472
Error	887	7,570,284

As a compromise to including all possible *GEI*_*ij*_'s in modeling, the all-interaction model and the regression on the mean model attempt to separate the contribution of GEI terms from the error. The strength of the regression on the mean model is in its ability to describe the GEI effect in terms of environmental effects, including through understanding the means of each environment. Next, the AMMI model uses principal components to remove the contribution of GEI from the error. Although only two principal components are represented here, more can be added to represent more possible complexity in interactions. However, if too many principal components are included, the error will approach zero and we are left with modeling that is equivalent to the all-interaction model. Unlike the all-interaction model, these models can be used to predict the performance of genotypes in untested environments.

The smallest SSEni acquired from our fitting no-interaction models on the final biclusters over 30 trials are 7,425,947, 9,602,964, and 7,405,088 for responses (2), (3), and (4), respectively. We can interpret this to mean that in terms of flowering time for sorghum, a linear model which captures the GEI and GEI + genotype average is more easily estimated than the other responses. Lastly, we are able to account for more GEI interaction than the regression on the mean and the AMMI models. Since we obtain smaller SSE and have the same main effects as the other four models, we can say that biclustering is able to simultaneously account for important interaction effects compared to the regression on the mean and the AMMI models.

### 3.2. Maize yield data

The next case was originally published in Ribaut et al. (1996) and Ribaut et al. (1997) and is used by Malosetti et al. (2013) to illustrate different models for phenotype predictions. The original authors crossed drought-tolerant and drought-susceptible parents, resulting in 211 maize (*Zea mays*) lines that were then subjected to stress trials over three separate years. This included managed water stress trials with no stress (1992), intermediate stress (1992 and 1994), several types of stress (1992 and 1994), and nitrogen stress trial with low and high nitrogen (1996), with a repeat of the low nitrogen stress trial in the same year, for a total of eight environments. The phenotypic response being considered is yield. In this section, and the following, we focus our discussion on the comparison of our biclustering model to the other linear models.

We immediately notice from Table 4 that the AMMI model with two principal components provides the model with the smallest error excluding the all-interaction model. Unlike the sorghum study, this data does not have appropriate numbers of genotype or environment clusters known in advance. Hence, we test multiple combinations of genotype and environment clusters. The results are found in Table 5. In summary, we see that by choosing three to four genotype clusters and three to four environmental clusters, we are able to account for more of the GEI effect. These clusters both provide smaller errors than the AMMI model and is a reasonable way to partition 211 genotypes and 7 environments due to the size of each cluster.

**Table 4:** Summary of degrees of freedom and sum of squares for the maize dataset.

**Term**	**Degrees of freedom**	**Sum of squares**
**No-interaction model**		
G	210	614
E	7	5,679
Error	1,470	813
**All-interaction model**		
G	210	614
E	7	5,679
GEI	1,470	813
Error	0	0
**Regression on mean model**		
G	210	614
E	7	5,679
GEI Ind	210	230
Error	1,260	583
**AMMI model**		
G	210	614
E	7	5,679
PC1	216	242
PC2	214	173
Error	1,040	398

**Table 5:** Biclustering results with the smallest SSE values obtained from fitting no-interaction models on each bicluster for raw responses (30 trials - maize data).

	**Response**		
**(G, E) - Pair**	**2**	**3**	**4**
(2, 2)	604	580	639
(2, 3)	418	463	446
(2, 4)	333	352	350
(3, 2)	579	595	524
(3, 3)	431	456	460
(3, 4)	300	339	314
(4, 2)	589	576	557
(4, 3)	434	454	402
(4, 4)	296	334	296

**Figure 4:**
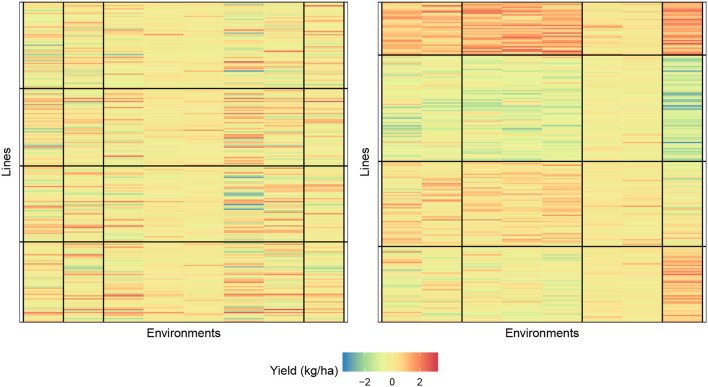
Biclustering illustration of response (4) with initialization **(left)** and final bicluster **(right)** for four row and column clusters (maize data). The homogeneity of the cells indicates that the biclustering algorithm was able to successfully group the response variables in distinct clusterings.

In Figure 4, we provide a visualization of the biclustering object with four genotypes and four environment clusters. The graphic on the left is the random initialization whereas the graphic on the right is the final bicluster result. We see that our methodology can indeed identify homogeneous blocks where useful interpretations can be made.

### 3.3. Rice flowering time data

The next case will be to consider a rice (*Oryza sativa*) dataset containing 176 lines and 9 environments. For each pair of lines and environments, we have two phenotypic measures for flowering time, FTgdd, and FTdap. FTgdd is the accumulated growing degree units for each line in an environment across the length of time the line was in that specific environment. Whereas, FTdap is day length for the line during its growth stage. Much like the previous section, we focus our discussion on the comparison of our biclustering model vs. the other linear model. Again since a study has not been performed on the optimal number of genotype and environment clusters, we aim to explore multiple combinations.

#### 3.3.1. Phenotype response FTgdd

The summary of our numerical results is displayed in Tables 6, 7. Similarly to our previous examples, the AMMI model is still superior to the no-interaction and regression on the mean models in being able to capture more GEI effects. In Table 7, we display the best results of our experimentation from 30 trials for various pairs of G and E with the metric being the SSE from fitting the no-interaction model after biclustering.

**Table 6:** Summary of degrees of freedom and sum of squares for the rice dataset—FTgdd.

**Term**	**Degrees of freedom**	**Sum of squares**
**No-interaction model FTgdd**		
G	175	132,865,164
E	8	19,169,848
Error	1355	59,620,672
**All-interaction model FTgdd**		
G	175	132,865,164
E	8	19,169,848
GEI	1355	59,620,672
Error	0	0
**Regression on mean model FTgdd**		
G	175	132,865,164
E	8	19,169,848
GEI Ind	175	23,653,430
Error	1180	35,967,242
**AMMI model FTgdd**		
G	175	132,865,164
E	8	19,169,848
PC1	182	50,094,094
PC2	180	3,362,842
Error	993	6,163,736

**Table 7:** Biclustering results with the smallest SSE values obtained from fitting no-interaction models on each bicluster for raw responses (30 trials—rice data FTgdd).

	**Phenotypic response**		
**(G, E) - Pair**	**2**	**3**	**4**
(7, 3)	4,605,916	4,692,904	4,606,188
(7, 4)	3,417,453	3,962,718	3,368,339
(8, 3)	4,604,048	4,653,856	4,503,244
(8, 4)	3,374,006	3,971,685	3,306,126
(9, 3)	4,577,429	4,385,709	4,460,673
(9, 4)	3,293,641	3,910,443	3,531,670

From Table 7, we see that we are able to obtain better numerical results than compared to the AMMI while still being able to cluster a reasonable number of lines and environments together. If seven row clusters are obtained, then in theory, each cluster would, on average, contain approximately 25 lines while sharing 2–3 environments. Consistently, responses 2 and 4 are able to obtain a lower SSEni than compared to other responses. Specifically, the interaction between the lines and environment is more easily identifiable than that of GEI + environmental average.

**Figure 5:**
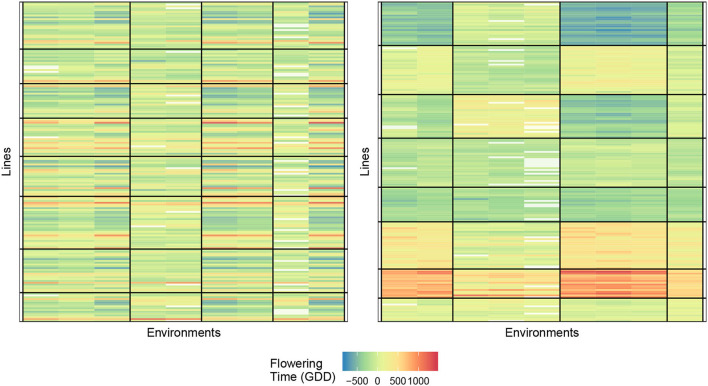
Biclustering illustration of response (4) with initialization **(left)** and final bicluster **(right)** for eight rows and four column clusters (rice data). The homogeneity of the cells indicates that the biclustering algorithm was able to successfully group the response variables in distinct clusterings.

Figure 5 displays the biclustering result of the initialization and final bicluster with eight lines and four environment clusters. We, again, notice the homogeneity of the blocks and the ability of our algorithm to identify meaningful groupings.

#### 3.3.2. Phenotype response FTdap

**Figure 6:**
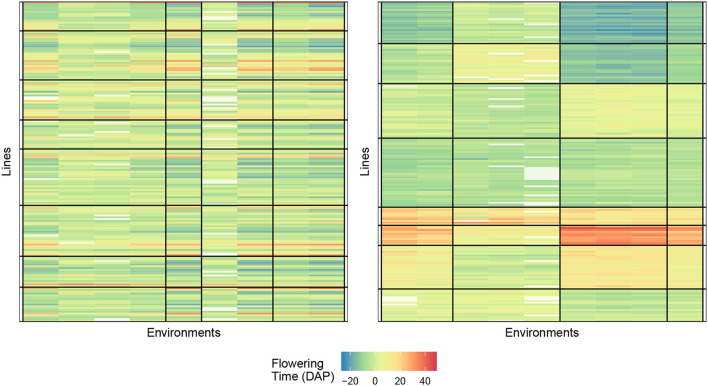
Biclustering illustration of response (4) with initialization **(left)** and final bicluster **(right)** for eight rows and four column clusters (rice data). The homogeneity of the cells indicates that the biclustering algorithm was able to successfully group the response variables in distinct clusterings.

The numerical results for FTdap are similar to FTgdd which can be seen in Tables 8, 9. That is, we are still able to account for more information than the regression on the mean and the AMMI model, given the correct combination. However, the most interesting observation is that Responses 2 and 4 are still consistently better than Responses 1 and 3. This is in line with the results shown for the Sorghum dataset. One possible explanation for this can be that the flowering time as a phenotypic trait is more easily identifiable with these responses. Recall that Response 2 is a direct expression for GEI and Response 4 is an expression for GEI + variety average. Figure 6 provides an illustration of the initial and final biclustering.

**Table 8:** Summary of degrees of freedom and sum of squares for the rice dataset—FTdap.

**Term**	**Degrees of freedom**	**Sum of squares**
**No-interaction model FTdap**		
G	175	134,189
E	8	485,135
error	1355	58,071
**All-interaction model FTdap**		
G	175	134,189
E	8	485,135
GEI	1355	58,071
Error	0	0
**Regression on mean model FTdap**		
G	175	134,189
E	8	485,135
GEI Ind	175	41,697
Error	1180	16,374
**AMMI model FTdap**		
G	175	134,405
E	8	484,918
PC1	182	47,971
PC2	180	4,272
Error	993	5,828

**Table 9:** Biclustering results with the smallest SSE values obtained from fitting no-interaction models on each bicluster for raw responses (30 trials—rice data FTdap).

	**Phenotypic response**		
**(G, E) - Pair**	**2**	**3**	**4**
(7, 3)	4,339	4,303	4,419
(7, 4)	3,535	3,511	3,362
(8, 3)	4,283	4,325	4,391
(8, 4)	3,471	3,502	3,505
(9, 3)	4,229	4,277	4,302
(9, 4)	3,406	3,467	3,252

## 4. Discussion

Recall that the primary goal of our biclustering is to obtain sets of varieties and environments where the GEI interaction effects are constant or equivalently zero within the frame of reference of each cell. If within a particular cell the GEI interaction effects are exactly the same for all of the varieties and all of the environments that define the cell, then no interaction effects are in fact observed within the cell. In other words, the phenotype of each variety in each environment can be predicted in terms of the main effects (genotypes and environment) only. Thus, for a perfect set of biclusters, the observations in each cell would follow such a no-interaction model. In light of this, we notice that indeed, our biclustering approach to modeling GEI interactions is indeed a valuable and novel approach.

For each of our three sample crops, we notice that a no-interaction model built from biclustering provides an appropriate fit, and in each case, we are able to account for more GEI than either the regression on the mean and the AMMI model from all four responses. Malosetti et al. (2013) provided the initial study on different approaches to modeling phenotypes given only genetics and environmental information. Our work has expanded upon their findings and results. Namely, we have shown an extension of their work by considering a successful biclustering technique to main effects modeling.

Admittedly, one limitation of this approach is the identification of the number of clusterings for the genetics and environment. Computationally, the number of clusterings can simply equal the dimensions of the dataset. However, in that case, we have a complete all-interaction model. Conversely, if we only have one genetic cluster and one environment cluster, we have a standard additive model. The difficulty lies in determining the optimal number of clusterings for each factor. Numerically, we have shown approaches to help aid in determining the number of clusterings while still maintaining strong interpretability. By incrementally increasing the number of clusterings along each factor, we can see the trade-off between complexity and SSE. Although still ambiguous, as the case with unsupervised machine learning, our biclustering approach provides a novel methodology to modeling genetics and environment data.

## 5. Conclusion

In this paper, we described a novel approach to modeling phenotypic data using a no-interaction model, that is, only incorporating the main effects of genotype (G) and environment (E). To accomplish this task, we made use of a biclustering algorithm to identify subsets of genotypes and subsets of environments where in this cell there exist no interaction effects. Because of the potential for phenotypic observations to be of missing, traditional statistical modeling methods cannot be used without imputing missing values which can bias data and results. Partially motivated by this, we utilized a novel biclustering algorithm that makes no assumptions on the completeness of data. This new algorithm enabled us to bicluster phenotypic observations when data is missing no-at-random which is similar to how most real-world plant breeding programs operate.

Our results showed that this approach is highly effective and out-performs the state-of-the-art linear models which only use phenotypic data as presented in Malosetti et al. (2013). In particular, we are able to obtain better performance than the regression on the mean and the additive main effects and multiplicative interactions model while still being able to interpret our findings. Along the way, we explained how to obtain a reasonable amount of clusters for the rows and columns and also explained the importance of first transforming the phenotypic response. This papers now aims to be the state-of-the-art when it comes to predicting phenotypic performance using only a two-way table of means consisting only of genotype and environment information.

## Data availability statement

The original contributions presented in the study are included in the article/Supplementary material, further inquiries can be directed to the corresponding author/s.

## Author contributions

HP and JR led the research and wrote the manuscript. SO and SV oversaw the research and edited the manuscript. AS contributed to the research idea and data processing. All authors contributed to the article and approved the submitted version.

## Funding

This research was supported in part by a Kingland Data Analytics Faculty Fellowship at Iowa State University.

## Conflict of interest

The authors declare that the research was conducted in the absence of any commercial or financial relationships that could be construed as a potential conflict of interest.

## Publisher's note

All claims expressed in this article are solely those of the authors and do not necessarily represent those of their affiliated organizations, or those of the publisher, the editors and the reviewers. Any product that may be evaluated in this article, or claim that may be made by its manufacturer, is not guaranteed or endorsed by the publisher.
